# Impact of the shared decision‐making process on lung cancer screening decisions

**DOI:** 10.1002/cam4.4445

**Published:** 2021-12-28

**Authors:** Naomi Q. P. Tan, Shawn P. E. Nishi, Lisa M. Lowenstein, Tito R. Mendoza, Maria A. Lopez‐Olivo, Laura C. Crocker, Karen R. Sepucha, Robert J. Volk

**Affiliations:** ^1^ Department of Health Services Research, Division of Cancer Prevention and Population Sciences The University of Texas MD Anderson Cancer Center Houston Texas USA; ^2^ Department of Internal Medicine, Division of Pulmonary Critical Care and Sleep Medicine The University of Texas Medical Branch Galveston Texas USA; ^3^ Department of Symptom Research, Division of Internal Medicine The University of Texas MD Anderson Cancer Center Houston Texas USA; ^4^ Division of General Internal Medicine Massachusetts General Hospital Harvard Medical School Boston Massachusetts USA

**Keywords:** cancer screening, decision aids, decision making, shared, implementation science, lung cancer

## Abstract

**Background:**

Professional organizations recommend the use of shared decision‐making (SDM) in supporting patients’ decisions about lung cancer screening (LCS). The objective of this study was to assess the impact of the SDM process on patient knowledge about LCS, decisional conflict, intentions to adhere to screening recommendations, and its role in how the patient made the final decision.

**Methods:**

This study surveyed patients screened for lung cancer within 12 months of the survey, recruited from two academic tertiary care centers in the South Central Region of the U.S. (May to July 2018).

**Results:**

Two hundred and sixty‐four patients completed the survey (87.9% White, 52% male, and mean age of 64.81). Higher SDM process scores (which indicates a better SDM process reported by patients) were significantly associated with greater knowledge of LCS (*b* = 0.17 *p* < 0.01). Higher SDM process scores were associated with less decisional conflict about their screening choice (*b* = 0.45, *p* < 0.001), greater intentions to make the same decision again (OR = 1.42, 95% CI = [1.06–1.89]), and greater intentions to undergo LCS again (OR = 1.32, 95% CI = [1.08–1.62]). The SDM process score was not associated with patients’ report of whether or not they shared the final decision with the healthcare provider (OR = 1.07, 95% CI = [0.85–1.35]).

**Conclusion(s):**

This study found that a better SDM process was associated with better affective‐cognitive outcomes among patients screened for lung cancer.

## Abstract 2

The impact of the shared decision‐making (SDM) process on patient outcomes in the context of lung cancer screening is understudied. This study found that a better SDM process was associated with greater knowledge of LCS, less decisional conflict, and intentions to adhere to LCS screening guidelines.

## 1 INTRODUCTION

In the U.S., lung cancer is the second most common cancer and the number one cause of cancer deaths among both men and women, accounting for about 22% of all deaths from cancer.^1^ The United States Preventive Services Task Force (USPSTF) recommends that high‐risk individuals undergo lung cancer screening (LCS).^2^ Given the harms and benefits of screening, shared decision‐making (SDM) is also recommended for LCS decisions.^3^, ^4^, ^5^ There is evidence that SDM can improve knowledge about LCS, including the benefits and harms of screening, increase interest in LCS, and reduce decisional conflict.^6^, ^7^, ^8^ The SDM process involves multiple steps including the physician offering options and describing risks and benefits, and patients expressing their values and preferences.^9^, ^10^ Even though SDM is conceptualized as having multiple components, the measurement of SDM in current research has largely focused on one part of the SDM process—the patients’ role in the final decision.^3^, ^11^ The impact of the overall SDM process, which include other components such as the discussion of risks and benefits or patient preferences, on outcomes has not been extensively researched. Hence, more research is needed to evaluate the impact of the overall SDM process on patient outcomes to strengthen the basis for implementation of SDM in clinical practice. In addition, the measurement of the quality of the SDM process is varied^12^ and scholars have called for greater consistency in the operationalization and measurement of the SDM process.^13^

Previous research suggests that are gaps in the use of SDM for LCS, such as inconsistent offering of LCS by providers, lack of discussion about the risks of LCS, and patients’ perception of limited SDM.^14^, ^15^ Hence, research examining the impact of SDM on health outcomes is important to promote greater use of SDM in LCS decisions. The primary objective of this study was to assess the impact of the SDM process on affective‐cognitive decision‐making outcomes, including knowledge of LCS, decisional conflict, intentions to adhere to screening recommendations, and whether they shared the final decision with their healthcare provider (HCP) or not (i.e., role in the final decision). A secondary objective was to evaluate the psychometric properties of the Shared Decision‐Making Process Survey^16^ in the context of LCS.

## 2 METHODS

We followed the reporting guidelines of The Strengthening the Reporting of Observational Studies in Epidemiology (STROBE) Statement for observational studies.^17^

### 2.1 Study design

This study used a survey with participants assessed at two time points, at baseline after receipt of an LCS test using low‐dose computed tomography (LDCT) and 1 month after the baseline survey. Additional details about the study methods and sample can be found elsewhere.^18^

### 2.2 Study setting

Recruitment of participants for this study took place at two academic tertiary care centers in the South Central Region of the United States. The first center is an academic medical center serving the South and Southeast Texas regions, while the second is a cancer care center located in a large metropolitan area also affiliated with a health system. The project was approved by the Institutional Review Board at both centers.

### 2.3 Participants and study size

Consecutive patients were invited to participate in this study by postal mail and/or email from May to July 2018. The inclusion criteria were patients (1) aged 55–80; (2) current or former smokers, and (3) screened for lung cancer using LDCT within 12 months of the survey. Participants from the academic medical center were referred for LCS during ambulatory encounters. Participants from the cancer center were recruited from two sources: (1) a tobacco treatment program in the cancer center; and (2) a LCS database that is part of the cancer center's LCS program. These participants had been referred for LCS by their primary care physician, cancer prevention center, pulmonary medicine, thoracic surgery, or thoracic oncology.

### 2.4 Measures

#### 2.4.1 Predictor

##### SDM process

The SDM process was measured using the Shared Decision‐Making Process survey^16^ (SDMP_4), one of the three performance measures of SDM process and outcome endorsed by the National Quality Forum.^19^ The SDMP_4 consisted of four items adapted to LCS: (1) discussion of options (whether the HCP explained that the patient had the option of not undergoing lung cancer screening; recoded as Yes = 1, No or Not sure = 0); (2) discussion of pros (the degree to which the HCP talked about the reasons the patient might want to be screened for lung cancer; recoded as A lot or Some = 1, A little or Not at all = 0); (3) discussion of cons (the degree to which the HCP talked about the reasons the patient might not want to be screened for lung cancer; recoded as A lot or Some = 1, A little or Not at all = 0); and (4) discussion of preferences (whether the HCP asked if the patient wanted to be screened for lung cancer; recoded as Yes = 1, No or Not sure = 0).

#### 2.4.2 Outcome variables

##### Affective‐cognitive outcomes

*Knowledge about LCS* was assessed using a sum of 16 items from a previous study,^20^ with higher scores indicating better knowledge of LCS (range of 0–16). *Decisional conflict*, or the individual's self‐reported level of comfort with the decision, was measured using SURE, a four‐item measure that was adapted from the Decisional Conflict Scale.^21^, ^22^ SURE is scored as a composite of four items ranging from 0 to 4, with higher scores indicating less decisional conflict.^21^ *Intentions to adhere to LCS screening recommendations* was measured using two items, the first asking whether patients would make the same decision again, and the second asking whether patients would undergo LCS again. Finally, *role in final decision* was measured using one item that asked whether patients made the final decision on their own, together with their healthcare provider, or if the healthcare provider made the final decision.

##### Demographics and other variables

Demographic variables included participants’ age, race, education level, smoking history, family history of lung cancer, LCS history, comorbidities, eligibility for LCS, and risk factors for lung cancer.

### 2.5 Quantitative variables

T1 measures (1 month after baseline) were only used to establish reliability. In all other analyses, T0 measures (baseline) were used and the data were examined cross‐sectionally. The SDM process scores were analyzed in two ways: (1) summed up to create a composite score ranging from 0 to 4, with higher scores indicating better quality of the SDM process,^16^ and (2) each of the four items used individually in the analyses to assess the *individual* impact of the conceptually distinct^23^ aspects of the SDM process on our outcomes. Covariates were determined a priori by entering gender and education level into a regression model with the outcome variables. Gender predicted likelihood of getting screened again, such that males were less likely to be screened compared to females (*b* = −0.21, *p* < 0.01). Education level was a significant predictor of T0 knowledge scores (*b* = 0.34, *p* < 0.001). Correlational analyses were carried out to assess any potential multicollinearity between variables (see Supplementary Material).

### 2.6 Statistical methods

The relationship between the SDM process (SDMP_4) and knowledge levels and decisional conflict was assessed with linear regression models, with education level as a covariate. Binary logistic regression models were used to assess the relationship between the SDM process (SDMP_4) and likelihood of making the same decision again, likelihood of being screened for lung cancer again, and likelihood of sharing the decision with their doctor. Education level and gender were included as covariates in logistic regression models.

To assess internal consistency of the SDMP_4, Cronbach's alpha was calculated using the recoded binary scores of the SDMP_4. In addition, the test‐retest reliability of the individual SDMP_4 items (original scores) was evaluated using Cohen's kappa. Finally, concurrent validity of the SDMP_4 was established through correlational analyses with CollaboRATE, another commonly used measure of SDM quality. All analyses were conducted in IBM SPSS Statistics Version 24.0, with *p* < 0.05 considered significant and 95% confidence intervals calculated for the odds ratios.

## 3 RESULTS

### 3.1 Sample characteristics

A total of 676 patients were assessed for eligibility; six were ineligible due to age, one due to smoking history, 43 were not invited as enrollment targets had been met. Among the 626 patients invited to participate, 4 refused and 356 did not respond. Please see elsewhere for a flow diagram of the recruitment process.^18^ The final sample (*N* = 264) was 52.0% male, with a mean age of 64.81. Majority (87.9%) of the participants were White, and 43.9% were college graduates.

### 3.2 SDM process scores

The overall SDM process score ranged from 0 to 4 (*M* = 2.19, *SD* = 1.34). The SDM process score had a slight negative skew and were mostly evenly distributed across the range of scores. Descriptive statistics of the individual SDMP_4 items have been reported elsewhere.^18^

### 3.3 Outcomes

#### 3.3.1 Knowledge

Overall knowledge levels were low among the sample (*M* = 6.58, *SD* = 2.75, range = 0–15). Please see Table 1 for a breakdown down of the mean knowledge score by levels of SDMP_4. Using the composite SDMP_4 score, we found that a higher SDM process scores were significantly associated with greater knowledge (*b* = 0.17, *p* < 0.01). Looking at the individual components of the SDM process, the findings showed that discussing the options (i.e., not being screened for lung cancer) (*b* = 0.16, *p* < 0.001), and the cons of LCS (*b* = 0.13, *p* < 0.05) were significantly associated with higher knowledge scores (Table 2). However, talking about the benefits of LCS and preferences for LCS were not associated with greater knowledge.

**TABLE 1:** Knowledge and decisional conflict scores by levels of overall SDM process scores (SDMP_4)

Levels of overall SDM process score	Knowledge^a^			Decisional conflict^b^		
	*N*	M	SD	*N*	M	SD
0	41	5.98	2.59	42	1.98	1.77
1	35	5.34	2.53	37	2.22	1.72
2	51	7.14	3.20	50	3.22	1.52
3	82	6.74	2.37	86	3.59	0.87
4	45	7.16	2.84	45	3.76	0.74

^a^ There were 16 knowledge items, recoded as either a 0 (Incorrect, Don't Know) or 1 (Correct), and summed up to create a composite knowledge score with a range of 0–15.

^b^ Decisional conflict was measured using four items in the SURE scale, recoded as either a 0 (No, Not Sure) or 1 (Yes), and summed up to create a composite decisional conflict score with a range of 0–4. Higher scores indicate less decisional conflict.

**TABLE 2:** Linear regression results of SDM process scores (SDMP_4) on knowledge and decisional conflict

Predictor/Outcome	Knowledge		Decisional conflict	
	*b*	*p*	*b*	*p*
Overall SDM process score	0.165	<0.01	0.449	<0.001
Edu. level	0.337	<0.001	0.060	0.28
	*R* ^2^ = 0.143		*R* ^2^ = 0.208	
Individual SDM process items				
Item 1: Options	0.160	<0.001	0.380	<0.001
Edu. level	0.328	<0.001	0.048	0.836
	*R* ^2^ = 0.141		*R* ^2^ = 0.150	
Item 2: Pros	0.085	0.154	0.437	<0.001
Edu. level	0.342	<0.001	0.077	0.172
	*R* ^2^ = 0.122		*R* ^2^ = 0.198	
Item 3: Cons	0.128	<0.05	0.222	<0.001
Edu. level	0.341	<0.001	0.084	0.170
	*R* ^2^ = 0.131		*R* ^2^ = 0.056	
Item 4: Preferences	0.115	0.054	0.274	<0.001
Edu. level	0.338	<0.001	0.072	0.231
	*R* ^2^ = 0.129		*R* ^2^ = 0.082	

Note

Predictors are binary variables (0 = No, 1 = Yes); knowledge scores range from 0 to 16; decisional conflict (measured by SURE scores) range from 0 to 4.

#### 3.3.2 Decisional conflict

Higher SDM process scores were associated with greater assuredness about their choice (*b* = 0.45, *p* < 0.001). Please see Table 1 for a breakdown down of the mean SURE score by levels of SDMP_4. In addition, individually, all aspects of the SDM process were significantly associated with greater assuredness about one's choice (i.e., less decisional conflict) (Table 2).

#### 3.3.3 Intentions to adhere to LCS screening recommendations

In terms of adherence intentions, 86.4% of the respondents indicated that they would definitely make the same decision again, 7.7% might, 3.8% might not, and 0.8% definitely would not. Overall, a better SDM process was positively associated with a greater likelihood of intending to make the same decision again (OR 1.42, 95% CI 1.06–1.89). With each unit increase in quality of the SDM process, the probability of participants intending to make the same decision again increased by 41.7%. Looking at the individual SDMP_4 items, the findings showed that discussing the pros of LCS was that only SDM component that was significantly associated with the likelihood intending to make the same decision again (OR 3.40, 95% CI 1.57–7.37), such that when the participant reported that the HCP talked about the pros of LCS, the probability that the patient would make the same decision again increased by 239.8%, compared to when the HCP did not.

In terms of undergoing LCS again, 66.7% of patients reported that they definitely would be screened again, 27.7% indicated “maybe”, and 4.2% indicated they would not. Overall, a better SDM process was indeed positively associated of intentions to undergo LCS again (OR 1.32, 95% CI 1.08–1.62). With each unit increase in quality of the SDM process, the probability of participants undergoing LCS again increased by 32.1%. Breaking down the individual SDMP_4 items, only discussing the pros of LCS (OR 1.97, 95% CI 1.11–3.48) and cons of LCS (OR 2.35, 95% CI 1.10–5.04) were significantly associated with the likelihood of intending to be screened again. When the patient reported that the HCP discussed the pros and cons of LCS, the probability that the patient would intend to undergo LCS again increased by 96.7% and 134.9% respectively. Please see Table 3 for the full results.

**TABLE 3:** Logistic regression results for the effect of SDM process scores (SDMP_4) on intentions to adhere and likelihood of sharing decision

	Make same decision again		Screen again		Shared decision (1) vs not shared (0)		Shared decision (1) vs made own decision (0)	
	OR	95% CI	OR	95% CI	OR	95% CI	OR	95% CI
Overall SDM process score	1.417*	1.063–1.888	1.321**	1.076–1.622	1.070	0.846–1.354	1.039	0.819–1.319
Individual SDM process items								
Item 1: Options	1.640	0.736–3.653	1.457	0.809–2.622	1.089	0.544–2.180	0.972	0.482–1.960
Item 2: Pros	3.398**	1.567–7.369	1.967*	1.110–3.484	1.481	0.742–2.957	1.401	0.699–2.808
Item 3: Cons	2.532	0.730–8.781	2.349*	1.095–5.041	1.108	0.527–2.329	1.061	0.504–2.234
Item 4: Preferences	1.502	0.703–3.207	1.712	0.990–2.960	0.994	0.531–1.859	0.949	0.506–1.782

Note

Each SDMP item was run as separate logistic regression models, with gender and education included as covariates in each model.

*p < 0.05; **p < 0.01.

### 3.4 Role in final decision

In terms of patients' role in the final decision, 74.6% of participants reported that they made the final decision on their own, 21.2% shared the decision with the HCP, and 2.3% said HCP made the final decision. This outcome was assessed two ways. First, we compared sharing the decision with the HCP versus not sharing (i.e., either made the final decision on their own or deferred the decision to the HCP). Second, given the small number of participants who deferred the decision to the HCP, we also compared participants who reported that they shared the decision with the HCP versus making the final decision on their own. Table 3 shows that neither the overall SDMP_4 score nor the individual SDMP_4 items, as well as education level and gender, were significant predictors of the participant sharing a decision with the HCP.

### 3.5 Psychometric evaluation of the SDMP_4

#### 3.5.1 Reliability

Overall, the SDMP_4 had acceptable internal consistency at both time points (T0: *M* = 2.19, *SD* = 1.34, α = 0.71; T1: *M* = 2.46, *SD* = 1.39, α = 0.74). Test‐retest reliability of overall composite SDMP_4 score was moderate (ICC = 0.61, *p* < 0.001).^24^ Test‐retest reliability of the individual SDMP_4 items was lower compared to the overall score (see Supplementary Materials).

#### 3.5.2 Validity

There was a moderate correlation between SDMP_4 and CollaboRATE at both time points (T0: *r* = 0.47, *p* < 0.001; T1: *r* = 0.47, *p* < 0.001).

## 4 DISCUSSION

This was one of the first studies to examine how the SDM process, measured using the Shared Decision‐Making Process (SDMP_4) survey, impacts short‐term, cognitive and behavioral outcomes, related to the decision to undergo LCS. Patients who reported higher SDM process scores were better informed about LCS and experienced less decisional conflict about their decision to undergo LCS. Furthermore, a better SDM process was also associated with greater intentions to adhere to the LCS recommendation for annual screening, suggesting lower levels of regret about their LCS decision. These findings strongly support the role SDM plays in leading to higher quality LCS decisions and potentially greater long‐term adherence. However, more research needs to be conducted to provide greater support for these findings.

We also found that a better SDM process was not significantly associated with whether the patient shared the final decision with their healthcare provider. Although majority of the patients in this sample made the final decision on their own, patients still reaped benefits from a better SDM process in terms of the outcomes. This suggests a conceptual distinction between SDM as a process and SDM as a description of how involved the patient was in the final decision, and the importance of measuring these concepts separately in future studies. In other words, a patient who chooses to defer the final decision to their provider can still have a good SDM process and want to be involved in the SDM process. A qualitative study found that about half of the sample distinguished between the shared process and the final decision, with participants expressing that even if there was a shared communication process leading up to the decision, the patient still took responsibility for making the final decision.^25^ It is possible that role preferences may be determined by other variables not measured in this study, such as health locus of control, which refers to one's belief about where control over their health lies.^26^, ^27^ This conceptual distinction may also explain mixed findings for the impact of SDM on outcomes in previous research.^3^

Our findings showed that different components of the SDM process were driving the effect on affective‐cognitive outcomes. Specifically, we found that discussing the options and risks of LCS predicted greater knowledge. One possible explanation is that most patients may be aware of the benefits of LCS given conventional wisdom that early detection is always best,^28^ but there may be knowledge gaps in terms of understanding that they have the option of not screening and that there are risks involved in LCS. In terms of decisional conflict, every aspect of the SDM process predicted how comfortable patients were with their screening decision. This is consistent with the notion that an SDM process that covers the key components that go into decision‐making should result in a more informed patient that is more comfortable with the final decision. Finally, we found that only discussing the pros of LCS was associated with making the same decision again, indicating that patients weigh benefits the most in terms of how comfortable they were with their decision. Discussing both the pros and cons of LCS was associated with intentions to be screened again, indicating that patients weigh both benefits and risks in their decision to be screened the following year. It appears that the informational aspects of the SDM process are most important for patients when it comes to intentions to adhere to LCS recommendations and that discussing cons of LCS did not deter patients. These findings highlight the importance of assessing the different components of the SDM process individually (i.e., discussion of options, pros, cons, and preferences using the SDMP_4^16^). Understanding which aspects of the SDM process have the greatest impact on patient outcomes has practical implications in a clinical setting where healthcare providers may not have resources to engage in every aspect of SDM with patients.

Finally, this study also assessed the psychometric properties of the Shared Decision‐Making Process survey (SDMP_4). The SDMP_4 demonstrated acceptable reliability and appeared to be a valid measure of the SDM process. The moderate correlation between the SDMP_4 and CollaboRATE was also expected given the slight difference in constructs measured. The SDMP_4 measures what was discussed during the SDM process (discussion of pros, cons, options, preferences), while CollaboRATE measures how much effort was spent on engaging patients (i.e., understanding health issues, listening to patient's concerns, and including patient choice in decision). Previous studies have already tested the SDMP_4 in other clinical settings (e.g., surgery decisions) and found it to be a robust measure.^23^ To the best of our knowledge, this study is the first to test the SDMP_4 for screening decisions, specifically LCS, further supporting the use of this measure across diverse medical contexts and patient populations. Future research should continue to test the SDMP_4 in a variety of settings to move toward a consistent measure of SDM quality.

This study had several limitations. First, this was a cross‐sectional survey, hence, our findings only show associations between variables. Second, we do not have information about the actual content of the SDM conversation between the patient and provider, for instance, what benefits or harms were discussed. Third, we did not collect information on patients’ comorbidities, which could have been used as a control variable in the analyses. In addition, the final sample was not fully representative of the general U.S. population as it was skewed toward a higher level of education. However, as initial contact with potential participants was done at screening sites, we did not have demographic information on individuals who opted not to participate in the study for comparison with the final sample. Finally, given that recruited patients had to have had LCS within 12 months of the survey, there may have been variations in how well the patients recalled the details of their SDM encounter. A previous manuscript analyzing the same data set found there was no statistically significant difference in knowledge, SDMP_4 scores, and decisional conflict between groups based on time since last LCS (comparing less than 3 months, 3–6 months, and 6–12 months).^18^ Hence, time since last screening was not included as a control in the analyses in this study.

In conclusion, this study found that a high quality SDM process may have benefits for patient knowledge, decisional conflict, and screening adherence, and provide support for a greater emphasis on improving the SDM process in the context of LCS in order to improve patient outcomes. This study also identified several theoretical implications for future research. First, the findings suggest that the individual components of the SDM process are conceptually distinct and have differential impact on outcomes. Second, the findings also suggest that patients may view the SDM process and their role in the final decision as distinct processes, and that patients who do not share the final decision with their HCP still benefit from a good SDM process in other ways.

## DISCLOSURE

No conflicts exist for the specified authors.

## AUTHOR CONTRIBUTIONS

All authors contributed to the conception of the paper, critically read and modified subsequent drafts, and approved the final version.

## ETHICS STATEMENT

This study was approved by the Institutional Review Boards at The University of Texas MD Anderson Cancer Center (PA14‐0072) and the University of Texas Medical Branch (18‐0084).

## Supporting information

Tables S1‐S2

## Data Availability

The data that supports the findings of this study are available in the supplementary material of this article.

## References

[1] American Cancer Society . Cancer Facts & Figures 2021 . https://www.cancer.org/content/dam/cancer‐org/research/cancer‐facts‐and‐statistics/annual‐cancer‐facts‐and‐figures/2021/cancer‐facts‐and‐figures‐2021.pdf Published 2021.

[2] U.S. Preventive Services Task Force . Lung Cancer: Screening . https://uspreventiveservicestaskforce.org/uspstf/recommendation/lung‐cancer‐screening#Table Published 2021, March 9

[3] Shay LA , Lafata JE . Where is the evidence? A systematic review of shared decision making and patient outcomes. Med Decis Making. 2015;35(1):114‐131. 25351843 10.1177/0272989X14551638 PMC4270851

[4] Stacey D , Légaré F , Boland L , et al. 20th anniversary Ottawa decision support framework: part 3 overview of systematic reviews and updated framework. Med Decis Making. 2020;40(3):379‐398. 32428429 10.1177/0272989X20911870

[5] Centers for Medicare & Medicaid Services . Decision memo for screening for lung cancer with low dose computed tomography (LDCT) (CAG‐00439N) . https://www.cms.gov/medicare‐coverage‐database/details/nca‐decision‐memo.aspx?NCAId=274 Published 2015, February 5

[6] Volk RJ , Linder SK , Leal VB , et al. Feasibility of a patient decision aid about lung cancer screening with low‐dose computed tomography. Prev Med. 2014;62:60‐63. 24518006 10.1016/j.ypmed.2014.02.006 PMC4593627

[7] Mazzone PJ , Tenenbaum A , Seeley M , et al. Impact of a lung cancer screening counseling and shared decision‐making visit. Chest. 2017;151(3):572‐578. 27815154 10.1016/j.chest.2016.10.027

[8] Fukunaga MI , Halligan K , Kodela J , et al. Tools to promote shared decision‐making in lung cancer screening using low‐dose ct scanning: a systematic review. Chest. 2020;158(6):2646‐2657. 32629037 10.1016/j.chest.2020.05.610 PMC7783863

[9] Makoul G , Clayman ML . An integrative model of shared decision making in medical encounters. Patient Educ Couns. 2006;60(3):301‐312. 16051459 10.1016/j.pec.2005.06.010

[10] Barry MJ , Edgman‐Levitan S . Shared decision making—The pinnacle patient‐centered care. N Engl J Med. 2012;336(9):780‐781. 10.1056/NEJMp1109283 22375967

[11] Degner LF , Sloan JA , Venkatesh P . The control preferences scale. Can J Nurs Res. 1997;21‐44. 9505581

[12] Gärtner FR , Bomhof‐Roordink H , Smith IP , Scholl I , Stiggelbout AM , Pieterse AH . The quality of instruments to assess the process of shared decision making: a systematic review. PLoS One. 2018;13(2):e0191747. 29447193 10.1371/journal.pone.0191747 PMC5813932

[13] Wollschläger D . Where is SDM at home? Putting theoretical constraints on the way shared decision making is measured. Z Evid Fortbild Qual Gesundhwes. 2012;106(4):272‐274. 22749074 10.1016/j.zefq.2012.04.004

[14] Lowenstein M , Vijayaraghavan M , Burke NJ , et al. Real‐world lung cancer screening decision‐making: barriers and facilitators. Lung Cancer. 2019;133:32‐37. 31200824 10.1016/j.lungcan.2019.04.026

[15] Redberg RF . Failing grade for shared decision making for lung cancer screening. JAMA Intern Med. 2018;178(10):1295‐1296. 30105372 10.1001/jamainternmed.2018.3527

[16] Sepucha KR & Fowler F . Shared Decision Making Process_4 User Guide v.1.0. Massachusetts General Hospital. https://mghdecisionsciences.org/wp‐content/uploads/2018/07/user‐guide‐sdmp_4‐nqf‐measure‐2962.pdf. Published 2018.

[17] Equator Network . The Strengthening the Reporting of Observational Studies in Epidemiology (STROBE) Statement: Guidelines for reporting observational studies . https://www.equator‐network.org/reporting‐guidelines/strobe/ Published 2021, April 13

[18] Nishi SPE , Lowenstein LM , Mendoza TR , et al. Shared decision‐making for lung cancer screening: how well are we “Sharing”? Chest. 2021;160(1):330‐340. 33556362 10.1016/j.chest.2021.01.041 PMC8295906

[19] National Quality Forum . National Quality Partners Shared Decision Making Action Brief . https://www.qualityforum.org/Publications/2017/10/NQP_Shared_Decision_Making_Action_Brief.aspx#:~:text=The%20National%20Quality%20Partners%20Shared,standard%20of%20person%2Dcentered%20care Published 2017, October

[20] Lowenstein LM , Richards VF , Leal VB , et al. A brief measure of smokers’ knowledge of lung cancer screening with low‐dose computed tomography. Prev Med Rep. 2016;4:351‐356. 27512650 10.1016/j.pmedr.2016.07.008 PMC4976139

[21] Ferron Parayre A , Labrecque M , Rousseau M , Turcotte S , Legare F . Validation of SURE, a four‐item clinical checklist for detecting decisional conflict in patients. Med Decis Making. 2014;34(1):54‐62. 23776141 10.1177/0272989X13491463

[22] Garvelink MM , Boland L , Klein K , et al. Decisional Conflict Scale use over 20 years: the anniversary review. Med Decis Making. 2019;39(4):301‐314. 31142194 10.1177/0272989X19851345

[23] Valentine K , Vo H , Fowler FJ Jr , Brodney S , Barry MJ , Sepucha KR . Development and evaluation of the shared decision making process scale: a short patient‐reported measure. Med Decis Making. 2021;41(2):108‐119. 33319648 10.1177/0272989X20977878

[24] Koo TK , Li MY . A guideline of selecting and reporting intraclass correlation coefficients for reliability research. J Chiropr Med. 2016;15(2):155‐163. 27330520 10.1016/j.jcm.2016.02.012 PMC4913118

[25] Shay LA , Lafata JE . Understanding patient perceptions of shared decision making. Patient Educ Couns. 2014;96(3):295‐301. 25097150 10.1016/j.pec.2014.07.017 PMC4241759

[26] Marton G , Pizzoli SFM , Vergani L , et al. Patients’ health locus of control and preferences about the role that they want to play in the medical decision‐making process. Psychol Health Med. 2020;26(2):260‐266. 10.1080/13548506.2020.1748211 32323553

[27] Hashimoto H , Fukuhara S . The influence of locus of control on preferences for information and decision making. Patient Educ Couns. 2004;55(2):236‐240. 15530760 10.1016/j.pec.2003.09.010

[28] Walsh‐Childers K , Braddock J . Competing with the conventional wisdom: newspaper framing of medical overtreatment. Health Commun. 2014;29(2):157‐172. 23537348 10.1080/10410236.2012.730173

