# Supplementary material for: Impact of the shared decision‐making process on lung cancer screening decisions
Source: Cancer Med. 2021 Dec 28;11(3):790–7. doi: 10.1002/cam4.4445 (PMC8817098; doi:10.1002/cam4.4445)
Supplement: Supplementary file 1 — Tables S1‐S2 [file CAM4-11-790-s001.docx]

**Supplementary Materials**

**Table 1:** Correlations between SDM Process scores and affective-cognitive outcomes

|  | SDMP_1 | SDMP_2 | SDMP_3 | SDMP_4 | Knowledge | Decisional conflict | Same decision | Screen again | Shared decision |
| --- | --- | --- | --- | --- | --- | --- | --- | --- | --- |
| SDMP_1^a^ | 1.00 | .48** | .25** | .46** | .18** | .38** | .09 | .09 | .01 |
| SDMP_2^a^ |  | 1.00 | .29** | .49** | .07 | .44** | .20** | .15* | .07 |
| SDMP_3^a^ |  |  | 1.00 | .29** | .12* | .22** | .10 | .15* | .01 |
| SDMP_4^a^ |  |  |  | 1.00 | .12 | .28** | .07 | .13* | .001 |
| Knowledge |  |  |  |  | 1.00 | .17** | .16* | .14* | .03 |
| SURE^b^ |  |  |  |  |  | 1.00 | .47** | .28** | -.10 |
| Same decision |  |  |  |  |  |  | 1.00 | .41** | -.12* |
| Screen again |  |  |  |  |  |  |  | 1.00 | .01 |
| Shared decision |  |  |  |  |  |  |  |  | 1.00 |

^a^SDMP_1 to SDMP_4 refer respectively to discussion of options, pros, cons, and preferences for shared decision making.

^b^Decision conflict was measured using SURE scores, such as greater scores indicate less decisional conflict.

**p* < .05, ***p* < .01

**Table 2:** Test-retest reliability of individual SDMP_4 items (Kappa and percent agreement)

| SDMP_4 Item | Kappa (κ) | % agreement |
| --- | --- | --- |
| Options | .34*** | 69.74% |
| Pros | .35*** | 54.39% |
| Cons | .31*** | 58.52% |
| Preferences | .53*** | 75.98% |

****p* < .001
